# Intrinsic detector sensitivity analysis as a tool to characterize ArcCHECK and EPID sensitivity to variations in delivery for lung SBRT VMAT plans

**DOI:** 10.1002/acm2.13221

**Published:** 2021-05-05

**Authors:** Thahabah Alharthi, Phil Vial, Lois Holloway, David Thwaites

**Affiliations:** ^1^ Institute of Medical Physics School of Physics The University of Sydney Sydney NSW Australia; ^2^ School of Medicine Taif University Taif Saudi Arabia; ^3^ Liverpool and Macarthur Cancer Therapy Centers Liverpool NSW Australia; ^4^ Ingham Institute for Applied Medical Research Sydney NSW Australia; ^5^ South Western Sydney Clinical School University of New South Wales Sydney NSW Australia; ^6^ Centre for Medical Radiation Physics University of Wollongong Wollongong NSW Australia

**Keywords:** ArcCHECK, EPID, Gamma analysis, intrinsic detector sensitivity, lung SBRT VMAT

## Abstract

**Purpose:**

To investigate intrinsic sensitivity of an electronic portal imaging device (EPID) and the ArcCHECK detector and to use this in assessing their performance in detecting delivery variations for lung SBRT VMAT. The effect of detector spatial resolution and dose matrix interpolation on the gamma pass rate was also considered.

**Materials and methods:**

Fifteen patients’ lung SBRT VMAT plans were used. Delivery variations (errors) were introduced by modifying collimator angles, multi‐leaf collimator (MLC) field sizes and MLC field shifts by ±5, ±2, and ±1 degrees or mm (investigating 103 plans in total). EPID and ArcCHECK measured signals with introduced variations were compared to measured signals without variations (baseline), using OmniPro‐I'mRT software and gamma criteria of 3%/3 mm, 2%/2 mm, 2%/1 mm, and 1%/1 mm, to test each system's basic performance. The measurement sampling resolution for each was also changed to 1 mm and results compared to those with the default detector system resolution.

**Results:**

Intrinsic detector sensitivity analysis, that is, comparing measurement to baseline measurement, rather than measurement to plan, demonstrated the intrinsic constraints of each detector and indicated the limiting performance that users might expect. Changes in the gamma pass rates for ArcCHECK, for a given introduced error, were affected only by dose difference (DD %) criteria. However, the EPID showed only slight changes when changing DD%, but greater effects when changing distance‐to‐agreement criteria. This is pertinent for lung SBRT where the minimum dose to the target will drop dramatically with geometric errors. Detector resolution and dose matrix interpolation have an impact on the gamma results for these SBRT plans and can lead to false positives or negatives in error detection if not understood.

**Conclusion:**

The intrinsic sensitivity approach may help in the selection of more meaningful gamma criteria and the choice of optimal QA device for site‐specific dose verification.

## 1 INTRODUCTION

The rapid development of advanced radiotherapy techniques such as intensity modulated radiotherapy (IMRT), volumetric modulated arc therapy (VMAT), and stereotactic body radiotherapy (SBRT) have enabled improved target dose conformity and optimization of dose to organs at risk.^1^, ^2^ However, their increased complexity requires careful understanding of the impact of potential errors and uncertainties on dose and also rigorous dose verification and patient‐specific quality assurance (QA) to ensure accurate treatment delivery.^1^, ^3^, ^4^ Several QA tools are commercially available for enabling dose evaluation by comparing planned dose distribution to the measured dose delivered.^3^, ^4^, ^5^ However, there remains limited and contradictory evidence regarding tolerances, suitability, and limitations of each QA device or system in the clinical setting for specific treatment sites, since it can be difficult to separate and evaluate the relative contribution of the different components of the system.^6^, ^7^, ^8^ The basic component is the intrinsic detector characteristics and their effect on the measured signal/dose (e.g., dose linearity, spatial resolution, signal‐to‐noise ratio (SNR), energy response, sensitivity to delivery variations). Next is any data correction or processing applied to the raw measured signal, used either to correct some of the intrinsic detector response issues, for example, to account for geometrical configuration and construction effects depending on detector system design, or in the dose calculation model employed by the system. Examples may include angular corrections in the ArcCHECK^9^ or calibration and correction factors used in the EPID fluence calculation to predict dose.^10^, ^11^ The third component is the model used to process the reference (planned) data to be compared to the processed measured data. This may be directly exported from the treatment planning system (TPS) as a dose matrix (direct TPS dose calculation), or from a separate calculation model that takes the TPS plan as input to calculate a predicted ‘dose’ signal onto an independent dose matrix representing the measurement distribution, as in some EPID models.^10^, ^12^ The last component comprises the dose comparison metrics such as the gamma index method,^6^ which combines dose difference (%DD) and distance to agreement (DTA) and has been widely accepted and implemented in clinical and commercial software. There are many configurable parameters, tolerances and different implementation methods, which in combination with the detector resolution and other characteristics can significantly affect the gamma results.^7^ Several studies have questioned the capability of various dosimetry systems and gamma analysis to detect clinically significant delivery errors.^8^, ^9^, ^10^, ^11^, ^12^ Whilst there are many published comparisons of clinical dosimetry systems, it is unclear how the results from any given system are driven by the intrinsic detector properties, as opposed to the other system components. The work presented here considers ArcCHECK and EPID as two examples with significantly different geometric characteristics. For each of these, recent relevant studies have evaluated the sensitivity to delivery errors, but have reached different conclusions regarding tolerances, detectability, and action levels.^13^, ^14^, ^15^, ^16^ For example, Woon et al.,^13^ and Maraghechi et al.,^14^ reported that an EPID was more sensitive than the ArcCHECK. However, Moliner et al.,^15^ found that the ArcCHECK sensitivity was higher than the EPID and that the combined use of the two detectors did not statistically improve error detectability compared to using one. Vieillevigne et al.,^16^ concluded that not all errors were detected and EPID and ArcCHECK showed similar sensitivity. All these studies have investigated head and neck (H&N) plans and/or prostate plans and had relatively small sample sizes (number of patients/baseline plans). They recommended different gamma criteria, which gives uncertainty in establishing action levels and consistent acceptance criteria.

Specifically, for lung VMAT SBRT plans there have been only limited studies in the literature. Hence, in our previous work we evaluated the sensitivity of both the ArcCHECK detector,^17^ and an EPID (Elekta iView GT),^18^ to a range of introduced delivery errors, using a conventional approach to test overall system sensitivity. That is, we used the standard procedure used for clinical QA, that is, in each case we compared measured doses, with and without errors, to the predicted values from the baseline (no error, NE) treatment plan.

In this study, we use a different approach, for an analysis of the intrinsic detector sensitivity properties. Here, we compare measured signals from plan deliveries with introduced errors to measured signals from the delivered baseline (no error) plan. That is, we compare detector signal to detector signal, rather than signal to plan‐predicted values. The aim is to eliminate a number of potentially confounding factors and to attempt to better understand the intrinsic performance of these dosimetry systems.

We propose that this can be used as a tool to test and characterize detectors’ intrinsic sensitivity and may represent a baseline optimal sensitivity that can be expected from any system utilizing a given detector type.

## 2 MATERIALS AND METHODS

### 2.A Plan selection and dose evaluation process

15 baseline lung SBRT VMAT plans (15 patients) and 88 generated plans with different delivery variations across these patients were selected from a previous planning study,^19^ based on the clinical significance of the differences. The plans were generated in the Pinnacle (Phillips Healthcare, Fitchburg, WI, USA), v9.8 treatment planning system (TPS), using a 6 MV photon beam from an Elekta Versa HD linear accelerator with a 40 leaf pairs Agility MLC (Elekta, Crawley, UK). For all patients, the plan consisted of two 200‐degree arcs with non‐zero collimator angle. VMAT plans were based on RTOG0236 and RTOG0915 planning guidelines.^20^, ^21^

Variations in delivery were introduced by modifying collimator angles by −5, −2, −1, +1, +2 or +5 degrees, by opening and closing MLC field sizes (MLCFS) and by introducing MLC shifts (MLCShift) of −5, −2, −1, +1, +2 or +5 mm. Any of these variations which caused any one or more of a range of DVH metrics to deviate by more than ±2% were defined as clinically significant. The following DVH metrics were considered; PTV (D_mean_, D_max_, V95%, V100%), spinal cord (D_mean_, D0.1 cc), and healthy lung (lung‐PTV) (D_mean_, V20 Gy).^19^ Only brief details were provided here of the planning study, to indicate the source of the selected plans, but full details can be seen in that reference.^19^ A total of 103 plans with different variations in delivery were selected for this study, where 15 were the baseline plans (no variations, or no errors, NE).

All lung SBRT VMAT baseline (NE) plans and the plans with introduced variations were exported to the Elekta Synergy Linac using the Mosaiq system (Elekta, Crawley, UK) for delivery. All measurements were those previously performed to test overall system sensitivity (measured doses vs expected/planned (TPS) doses).^17^, ^18^

One of the NE patient plans was selected as a consistent benchmark test to assess the level of measurement consistency over the period during which the measurements were performed. This test was performed before running each set of measurements to ensure that the plans passed the global gamma analysis with 3%/3 mm, 2%2 mm, 2%/1 mm, 1%/1 mm tolerance criteria. In addition, the measured baseline plan was compared for each measurement session to the measured plans for both ArcCHECK and EPID detectors in this study.

### 2.B Detectors

#### 2.B.1 ArcCHECK

The ArcCHECK (Sun Nuclear Corporation (SNC), USA) is a cylindrical phantom consisting of 1386 n‐Si diode detectors arranged in a helical array with 1 cm detector spacing. The diodes are positioned within an outer rim with dimensions of 21 cm length, 20.8 cm external diameter and 15.1 cm internal diameter with a central cylindrical replaceable insert.^9^ The ArcCHECK detector was calibrated and used for the measurements based on the manufacturer’s recommendation (more details on these measurements can be found in our previous ArcCHECK experimental study).^17^

#### 2.B.2 EPID

The EPID (an amorphous silicon EPID; Elekta iView GT) has a sensitive area of 41 cm × 41 cm in size, consisting of 1024 × 1024 pixels of 0.4 × 0.4 mm^2^ and a nominal source‐detector distance of 160 cm.^22^, ^23^ The measured EPID images were converted into dose matrices using a calibration procedure and an existing methodology primarily developed by Lee et al.,^24^, ^25^ and Matlab computer code outlined elsewhere.^26^ (more details can be found in our previous EPID experimental study).^18^

### 2.C Intrinsic detector sensitivity dose matrix analysis

The analysis presented here compared the EPID and ArcCHECK measured signal values for plans with introduced delivery variations to those for the same detector without variations (baseline), using OmniPro I’mRT (IBA Dosimetry, V1.6) software. An in‐house Python code was used to convert ArcCHECK dose matrix files into OmniPro‐I'mRT software compatible file format for the intrinsic detector sensitivity analysis. First, ArcCHECK measured dose matrices with introduced delivery variations were compared to the measured baseline ArcCHECK dose matrices (ArcCHECK signal vs ArcCHECK _NE_ signal) and then the EPID measured dose matrices with variations in delivery were compared to the measured baseline EPID dose matrices (EPID signal vs EPID_NE_ signal) using the gamma index method.^6^

The gamma analysis was performed using global (G) dose difference (DD%) and distance‐to‐agreement (DTA) criteria with tolerances of 3%/3 mm, 2%/2 mm, 2%/1 mm, and 1%/1 mm. The dose threshold was selected to be for dose points receiving greater than or equal to 10% of the maximum dose for both ArcCHECK and EPID analysis.

The ArcCHECK and EPID measured dose distributions were left at their native (original) re‐sampled resolution of 0.5 and 0.0255 cm respectively. In total, 206 plans were compared, and 824 analyses were performed. The average gamma pass rate (GPR) was determined as the percentage of assessed points that have a gamma score of less than or equal to 1. The gamma mean value (GMV) was also calculated, as the mean of the gamma scores of all assessed points for each dose matrix comparison for both ArcCHECK and EPID. The GMV was used to support the gamma pass rate assessment, where decreased GMV should correlate with increased gamma pass rate.

### 2.D ArcCHECK field‐by‐field (individual arc) measurements vs composite plan measurements

This study was based on the data taken in our previous published work using an ArcCHECK detector^17^ with a plan‐based approach (composite arcs/whole plan measurements) and using an EPID^18^ with a field (arc)‐based approach. In each case these approaches were followed, as those common in clinical practice for these devices. To assess the impact of the different approaches as a potential confounding factor and to minimize bias in the comparison of findings for the two detectors, a subset of the ArcCHECK measurements (37 plans: 5 original/baseline plans + 32 plans with errors) were repeated for individual arcs and the findings compared to the composite measurements. Similar analysis as above, with the same criteria using gamma pass rate and GMV, were performed using OmniPro I’mRT 1‐7 (IBA Dosimetry, v 1.7) software.

### 2.E Assessment of the detectors (dose matrix) resolution and interpolation effect on the gamma analysis

To further validate the results and to quantify the effect of the dose matrix resolution and its interpolation on the gamma index, a random sample was selected (seven patients; with the associated 43 plans with different variations in delivery) and the dose matrix (sampling) resolution was changed to 1 mm for both ArcCHECK and EPID. Gamma analysis was used, with the same tolerances, to compare ArcCHECK and EPID measured plans with introduced variations in delivery to those without variations (baseline) using OmniPro‐I'mRT software.

## 3 RESULTS

The repeated benchmark baseline plan check measurements were generally consistent throughout. When comparing repeated measured baseline (no error) plans to each other, for either ArCHECK or EPID, the gamma pass rate was consistent (at 100%) and this was considered as the standard NE plans pass rate, with negligible uncertainty. The measured deliveries with introduced errors were compared relative to the NE measured results and errors were considered detected if the pass rate is less than for that standard NE measurement to measurement value.

**Fig. 1:**
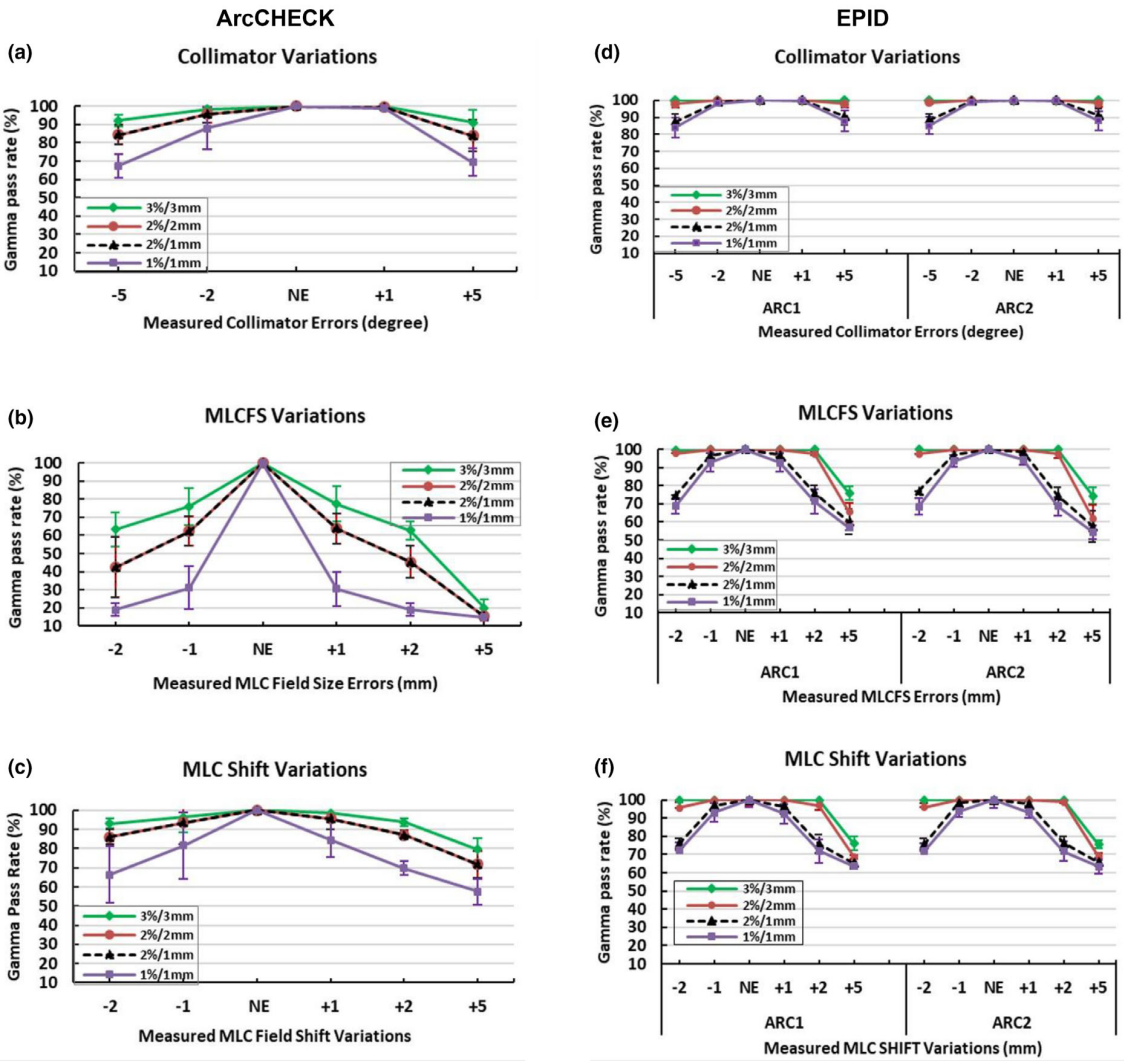
Detector intrinsic sensitivity expressed as the gamma pass rate for the measured dose distribution with introduced‐variations in delivery vs measured baseline plans with no variation/no error (NE). Average gamma pass rate of the baseline (NE) plans and those with introduced variations in collimator angles and MLC using ArcCHECK, whole composite plan (left column; a–c) and EPID; individual arc measurement ARC1 and ARC2 (right column; d–f) with different gamma criteria. The error bars represent the standard deviation (±1SD) of the measured plans. Correction added on May 28, 2021 after first online publication: Figure columns have been updated as 'ArcCHECK' (above left column: a‐c) and 'EPID' (above right column : d‐f).

Figure 1 shows the detector intrinsic sensitivity represented as mean global gamma pass rates of the measured dose matrices for the delivered plans with the introduced variations when compared to the same measurements for the delivered NE plans for both ArcCHECK and EPID, using the four gamma criteria of 3%/3 mm, 2%/2 mm, 2%/1 mm, and 1%/1 mm. Overall, the behavior of the gamma pass rate, decreasing as the magnitude of the variations increased, was as expected. For all delivery variation types (collimator angle, ±MLCFS, and ±MLC Shift), the intrinsic sensitivities of the ArcCHECK and EPID in detecting the introduced variations depended significantly on the gamma tolerance setting, with more variations detected with tighter gamma criteria. All simulated MLCFS and MLC Shift and ±5‐degree collimator variations can be detected using specific gamma tolerances for each variation magnitude as shown in Fig. 1, Tables 1 and 2. Collimator variations of +1 and −2 degree showed no significant change in the gamma pass rate when compared to the measured NE plan for all gamma criteria for both ArcCHECK and EPID.

**Table 1:** Mean (±1SD) gamma pass rate difference between the original ArcCheck dose matrix resolution of 5 mm (left columns) and 1mm resolution (right column) for lung SABR VMAT plans delivered with different collimator, MLCFS and MLCShift variations. A lower gamma pass rate number indicates greater sensitivity to the introduced‐variation.

Variation type/magnitude	Gamma pass rate (%, mean ± SD) ArcCheck original image resolution of (5 mm)				Gamma pass rate (%, mean ± SD) ArcCheck image resolution of (1 mm)			
	3%/3 mm	2%/2 mm	2%/1 mm	1%/1 mm	3%/3 mm	2%/2 mm	2%/1 mm	1%/1 mm
Collimator (degree)								
+1°	99.68 ± 0.0	99.44 ± 0.0	99.44 ± 0.0	98.84 ± 0.0	99.97 ± 0.0	99.84 ± 0.0	99.61 ± 0.0	99.04 ± 0.0
+5°	91.53 ± 4.9	82.97 ± 7.2	82.97 ± 7.2	67.93 ± 6.3	99.06 ± 0.1	93.55 ± 2.5	84.23 ± 7.1	69.83 ± 7.6
−2°	99.40 ± 0.0	99.07 ± 0.0	99.07 ± 0.0	96.19 ± 0.0	99.96 ± 0.0	99.67 ± 0.0	99.21 ± 0.0	96.98 ± 0.0
−5°	92.96 ± 3.9	86.01 ± 6.5	86.01 ± 6.5	70.59 ± 4.7	98.38 ± 1.0	93.50 ± 3.1	87.20 ± 6.9	72.36 ± 5.7
MLC field size (mm)								
+1	78.87 ± 8.5	64.01 ± 8.6	64.01 ± 8.6	30.27 ± 10.8	94.08 ± 5.3	79.53 ± 7.3	64.61 ± 8.3	30.17 ± 11.1
+2	65.20 ± 0.0	46.36 ± 0.0	46.36 ± 0.0	16.89 ± 0.0	91.54 ± 0.0	66.06 ± 0.0	46.74 ± 0.0	16.52 ± 0.0
+5	23.70 ± 0.0	16.03 ± 0.0	16.03 ± 0.0	15.89 ± 0.0	41.34 ± 0.0	21.94 ± 0.0	15.54 ± 0.0	15.44 ± 0.0
−1	75.91 ± 8.4	60.67 ± 4.2	60.67 ± 4.2	28.18 ± 4.8	92.53 ± 5.1	77.67 ± 4.3	61.48 ± 3.7	28.28 ± 4.9
−2	56.69 ± 0.0	30.74 ± 0.0	30.74 ± 0.0	16.40 ± 0.0	74.26 ± 0.0	45.93 ± 0.0	30.73 ± 0.0	16.17 ± 0.0
MLC shift (mm)								
+1	98.02 ± 2.7	94.49 ± 6.6	94.49 ± 6.6	82.79 ± 10.5	99.85 ± 0.1	98.62 ± 1.8	95.06 ± 6.8	84.09 ± 10.9
+2	94.74 ± 0.0	87.39 ± 0.0	87.39 ± 0.0	70.55 ± 0.0	99.25 ± 0.0	94.31 ± 0.0	88.21 ± 0.0	73.03 ± 0.0
+5	75.39 ± 0.0	66.82 ± 0.0	66.82 ± 0.0	52.53 ± 0.0	91.78 ± 0.0	80.91 ± 0.0	68.50 ± 0.0	53.82 ± 0.0
−1	94.68 ± 9.3	90.95 ± 13.7	90.95 ± 3.7	77.81 ± 21.3	97.66 ± 4.5	94.89 ± 9.2	91.51 ± 13.8	79.07 ± 21.1
−2	92.84 ± 2.7	85.25 ± 3.6	85.25 ± 3.6	70.20 ± 3.8	98.90 ± 0.9	93.89 ± 1.6	86.48 ± 4.2	71.58 ± 4.1

**Table 2:** Mean (±1SD) gamma pass rate difference between the original EPID dose image resolution of 0.255 mm (left columns) and 1mm resolution (right column) for lung SABR VMAT plans delivered with different collimator, MLCFS and MLCShift variations. Each plan consisted of two Arcs (Arc1 and Arc2). A lower gamma pass rate number indicates greater sensitivity to the introduced‐variation.

Variation type/magnitude		Gamma pass rate (%, mean ± SD) EPID original image resolution of (0.255 mm)				Gamma pass rate (%, mean ± SD) EPID image resolution of (1 mm)			
		3%/3 mm	2%/2 mm	2%/1 mm	1%/1 mm	3%/3 mm	2%/2 mm	2%/1 mm	1%/1 mm
Collimator (degree)									
+1°	Arc1	100.0 ± 0.0	100.0 ± 0.0	99.98 ± 0.0	99.66 ± 0.0	99.50 ± 0.0	97.37 ± 0.0	94.27 ± 0.0	88.51 ± 0.0
	Arc2	100.0 ± 0.0	100.0 ± 0.0	100.0 ± 0.0	99.67 ± 0.0	99.64 ± 0.0	97.67 ± 0.0	88.88 ± 0.0	83.80 ± 0.0
+5°	Arc1	100.0 ± 0.0	99.87 ± 0.1	93.27 ± 3.0	90.73 ± 2.4	99.64 ± 0.3	97.67 ± 2.0	88.88 ± 6.8	83.80 ± 8.6
	Arc2	100.0 ± 0.0	99.90 ± 0.1	92.65 ± 2.5	90.85 ± 2.3	99.48 ± 0.1	95.67 ± 1.9	85.83 ± 6.2	81.47 ± 7.7
−2°	Arc1	100.0 ± 0.0	100.0 ± 0.0	99.42 ± 0.0	98.88 ± 0.0	99.64 ± 0.0	96.32 ± 0.0	89.21 ± 0.0	83.87 ± 0.0
	Arc2	100.0 ± 0.0	100.0 ± 0.0	99.34 ± 0.0	98.65 ± 0.0	99.64 ± 0.0	96.47 ± 0.0	89.16 ± 0.0	83.61 ± 0.0
−5°	Arc1	100.0 ± 0.0	98.82 ± 1.3	89.85 ± 1.3	87.17 ± 2.3	99.31 ± 0.2	92.43 ± 1.1	79.64 ± 1.6	73.98 ± 3.5
	Arc2	100.0 ± 0.0	99.08 ± 1.3	89.75 ± 3.7	87.40 ± 4.6	99.28 ± 0.4	92.91 ± 1.6	79.63 ± 4.2	73.95 ± 6.3
MLCFS (mm)									
+1	Arc1	100.0 ± 0.0	100.0 ± 0.0	96.77 ± 0.9	92.51 ± 1.8	99.47 ± 0.3	96.90 ± 0.8	77.32 ± 3.2	73.85 ± 4.3
	Arc2	100.0 ± 0.0	100.0 ± 0.0	98.13 ± 0.9	93.83 ± 1.7	99.68 ± 0.2	97.43 ± 0.9	77.62 ± 3.6	74.43 ± 4.4
+2	Arc1	100.0 ± 0.0	97.95 ± 0.0	79.02 ± 0.0	76.18 ± 0.0	99.32 ± 0.0	85.82 ± 0.0	74.12 ± 0.0	70.70 ± 0.0
	Arc2	99.96 ± 0.0	98.81 ± 0.0	78.41 ± 0.0	74.30 ± 0.0	99.99 ± 0.0	86.85 ± 0.0	73.74 ± 0.0	68.76 ± 0.0
+5	Arc1	81.36 ± 0.0	68.76 ± 0.0	64.05 ± 0.0	60.61 ± 0.0	78.08 ± 0.0	67.12 ± 0.0	63.70 ± 0.0	59.87 ± 0.0
	Arc2	79.12 ± 0.0	67.73 ± 0.0	64.52 ± 0.0	61.33 ± 0.0	75.77 ± 0.0	67.15 ± 0.0	63.84 ± 0.0	59.36 ± 0.0
−1	Arc1	100.0 ± 0.0	100.0 ± 0.1	96.39 ± 1.2	92.47 ± 1.9	99.33 ± 0.5	96.55 ± 1.2	77.71 ± 3.6	73.63 ± 3.6
	Arc2	100.0 ± 0.0	99.92 ± 0.2	96.14 ± 3.0	92.84 ± 2.9	99.62 ± 0.2	97.09 ± 1.0	77.92 ± 4.0	74.32 ± 4.1
−2	Arc1	100.0 ± 0.0	96.36 ± 0.0	79.75 ± 0.0	75.10 ± 0.0	99.53 ± 0.0	84.24 ± 0.0	75.71 ± 0.0	69.64 ± 0.0
	Arc2	100.0 ± 0.0	96.62 ± 0.0	78.67 ± 0.0	73.98 ± 0.0	95.56 ± 0.0	84.56 ± 0.0	72.50 ± 0.0	66.73 ± 0.0
MLC shift (mm)									
+1	Arc1	100.0 ± 0.0	100.0 ± 0.0	95.07 ± 4.3	91.13 ± 5.4	99.46 ± 0.4	96.64 ± 1.1	77.97 ± 3.6	74.62 ± 4.0
	Arc2	100.0 ± 0.0	100.0 ± 0.0	96.94 ± 0.7	91.83 ± 2.5	99.75 ± 0.2	97.54 ± 0.9	78.54 ± 3.9	75.52 ± 4.3
+2	Arc1	100.0 ± 0.0	98.39 ± 0.0	73.63 ± 0.0	69.23 ± 0.0	99.61 ± 0.0	82.64 ± 0.0	69.06 ± 0.0	64.21 ± 0.0
	Arc2	100.0 ± 0.0	99.21 ± 0.0	75.86 ± 0.0	70.08 ± 0.0	99.43 ± 0.0	85.14 ± 0.0	69.32 ± 0.0	63.25 ± 0.0
+5	Arc1	78.64 ± 0.0	69.26 ± 0.0	64.90 ± 0.0	62.71 ± 0.0	75.92 ± 0.0	67.99 ± 0.0	64.79 ± 0.0	62.09 ± 0.0
	Arc2	77.09 ± 0.0	67.79 ± 0.0	64.21 ± 0.0	60.75 ± 0.0	74.64 ± 0.0	67.18 ± 0.0	63.89 ± 0.0	60.09 ± 0.0
−1	Arc1	100.0 ± 0.0	100.0 ± 0.0	97.50 ± 3.6	94.70 ± 5.0	99.43 ± 0.0	96.33 ± 0.6	78.51 ± 3.2	74.83 ± 4.4
	Arc2	100.0 ± 0.0	100.0 ± 0.0	97.36 ± 1.8	92.19 ± 1.7	99.66 ± 0.2	97.34 ± 1.0	78.72 ± 4.4	75.67 ± 4.9
−2	Arc1	100.0 ± 0.0	94.70 ± 3.5	74.87 ± 2.0	70.72 ± 2.8	99.69 ± 0.0	82.13 ± 1.0	71.50 ± 2.8	67.50 ± 3.6
	Arc2	100.0 ± 0.0	94.20 ± 3.2	75.90 ± 0.9	72.26 ± 1.6	99.58 ± 0.4	82.41 ± 1.0	72.37 ± 1.8	67.46 ± 3.5

For the same gamma criteria, the effect of the detector resolution on the gamma index analysis for these SBRT deliveries, and the limitations of the ArcCHECK and EPID, were clearly seen in Fig. 1. In the case of ArcCHECK, the dose difference (DD %) criteria dominated the gamma results. For example, the gamma pass rates were the same when using either 2%/2 mm or 2%/1 mm for all delivery variation types and magnitudes. Changes in the gamma pass rates were only seen when changing the DD% from 3% to 2% or 1%. In contrast, the EPID gamma results showed only slight changes when changing the DD% metric, but more effect when changing distance to agreement (DTA) criteria., for example, when changing DTA from 2 to 1 mm (Fig. 1).

**Fig. 2:**
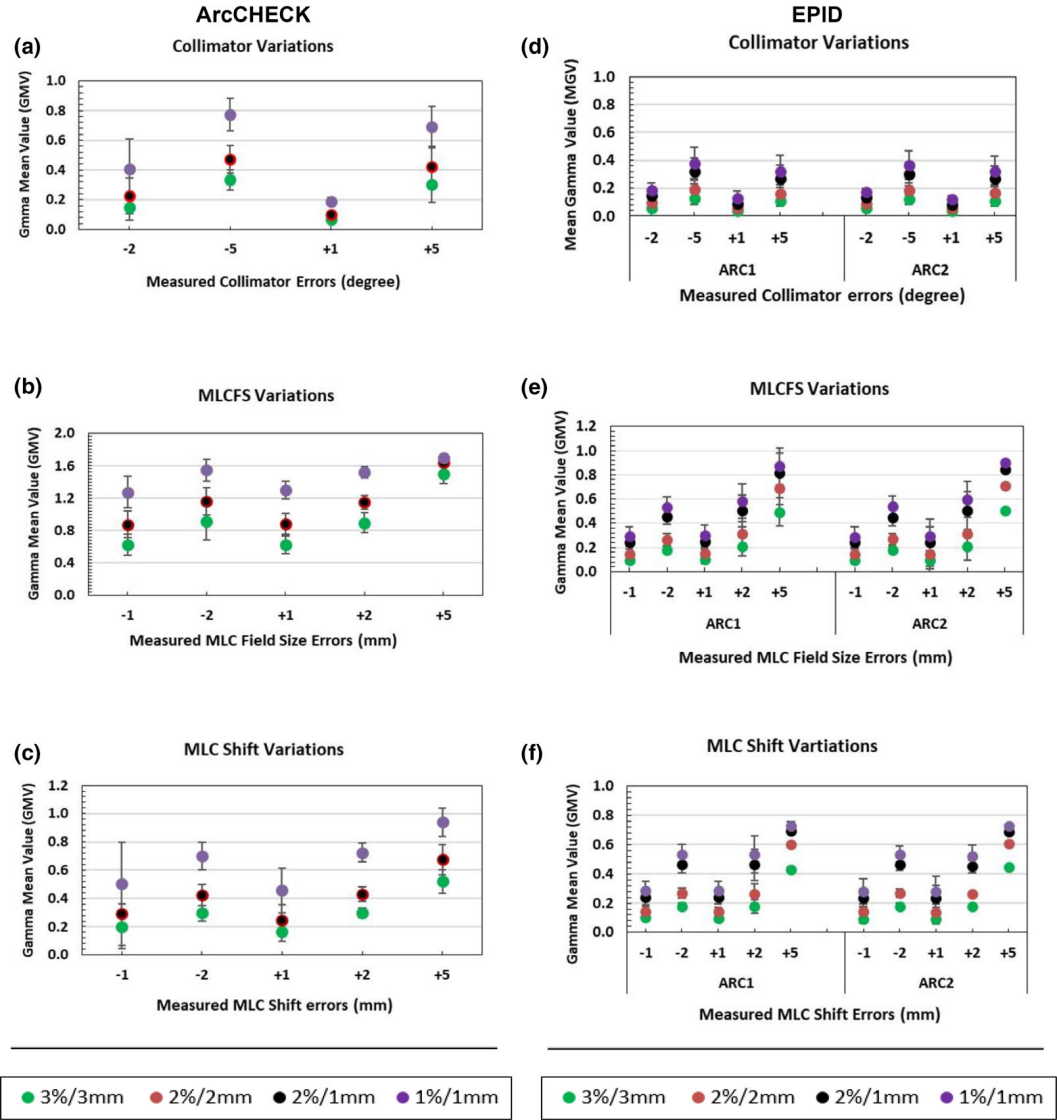
Detector intrinsic sensitivity expressed as the gamma mean values (GMV) of the measured dose distributions with introduced variations in delivery. Average mean gamma value of simulated variations in collimator and MLC when using ArcCHECK, whole composite plan (left column; a–c) and EPID; individual arc measurement ARC1 and ARC2 (right column; d–f) with different gamma criteria. The error bars represent the standard deviation (±1SD). Correction added on May 28, 2021 after first online publication: Figure columns have been updated as 'ArcCHECK' (above left column: a‐c) and 'EPID' (above right column : d‐f).

The gamma pass rate (GPR) results (Fig. 1) were generally further supported from the corresponding gamma mean value (GMV) results (Fig. 2). For all delivery variation types, the GMV increased as the variation magnitude increased, as expected, and stricter gamma criteria had higher GMV for the same variation type. Additionally, GMV were similar for the ArcCHECK when using the same DD% of 2%/2 mm and 2%/1 mm, but different for the EPID as the DTA changed, as shown in Fig. 2.

**Fig. 3:**
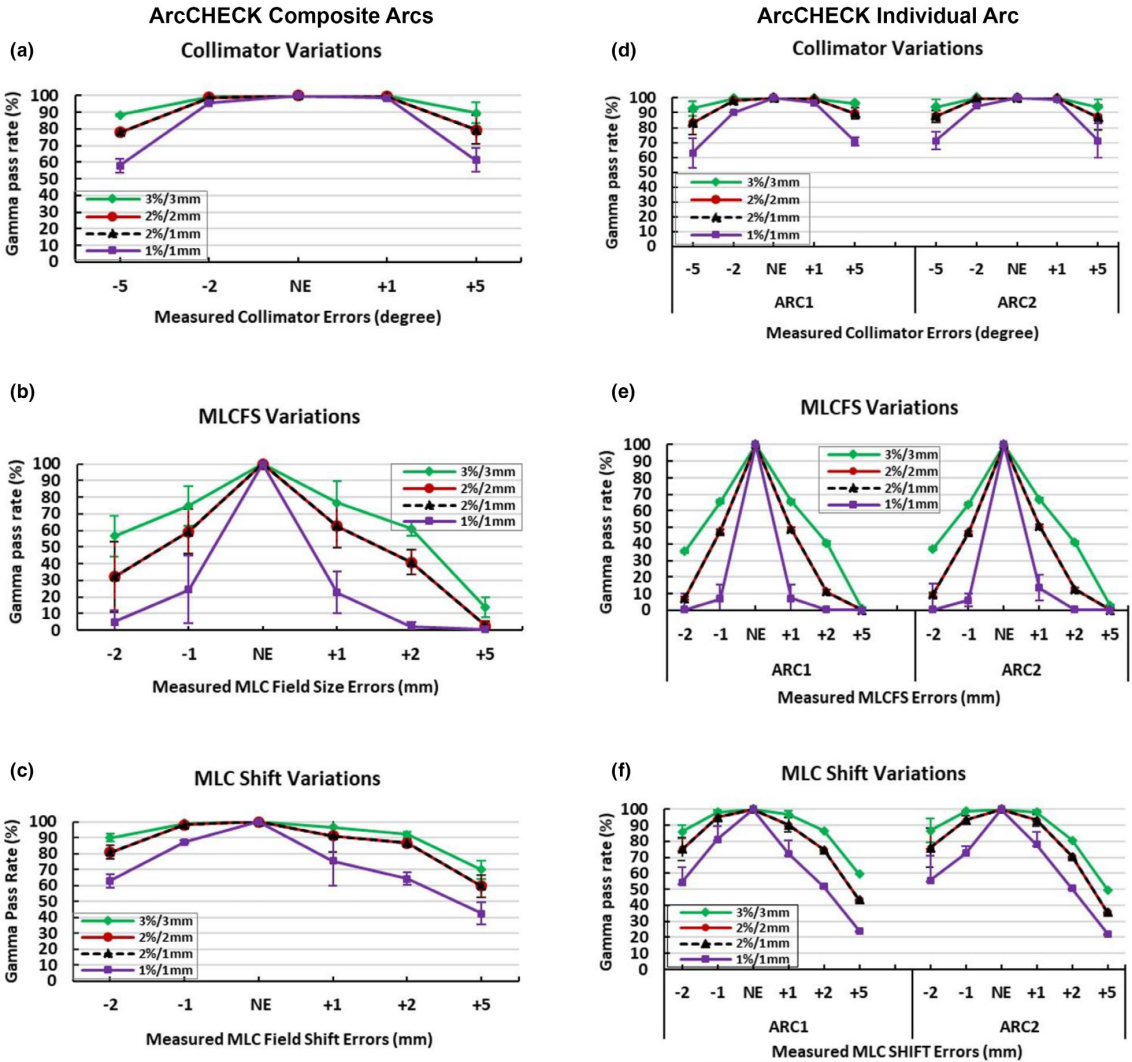
Detector intrinsic sensitivity expressed as the gamma pass rate for the measured dose distribution with introduced‐variations in delivery vs measured baseline plans with no variation/no error (NE). Average gamma pass rate of the baseline (NE) plans and simulated variations in collimator angles and MLC using ArcCHECK; composite arcs/plan measurements (left column; a–c) and ArcCHECK; individual arc measurement ARC1 and ARC2 (right column; d–f) with different gamma criteria. The error bars represent the standard deviation (±1SD) of the measured plans. Correction added on May 28, 2021 after first online publication: Figure columns have been updated as ArcCHECK Composite Arcs (above left column: a‐c) and ArcCHECK Individual Arc (above right column: d‐f).

**Fig. 4:**
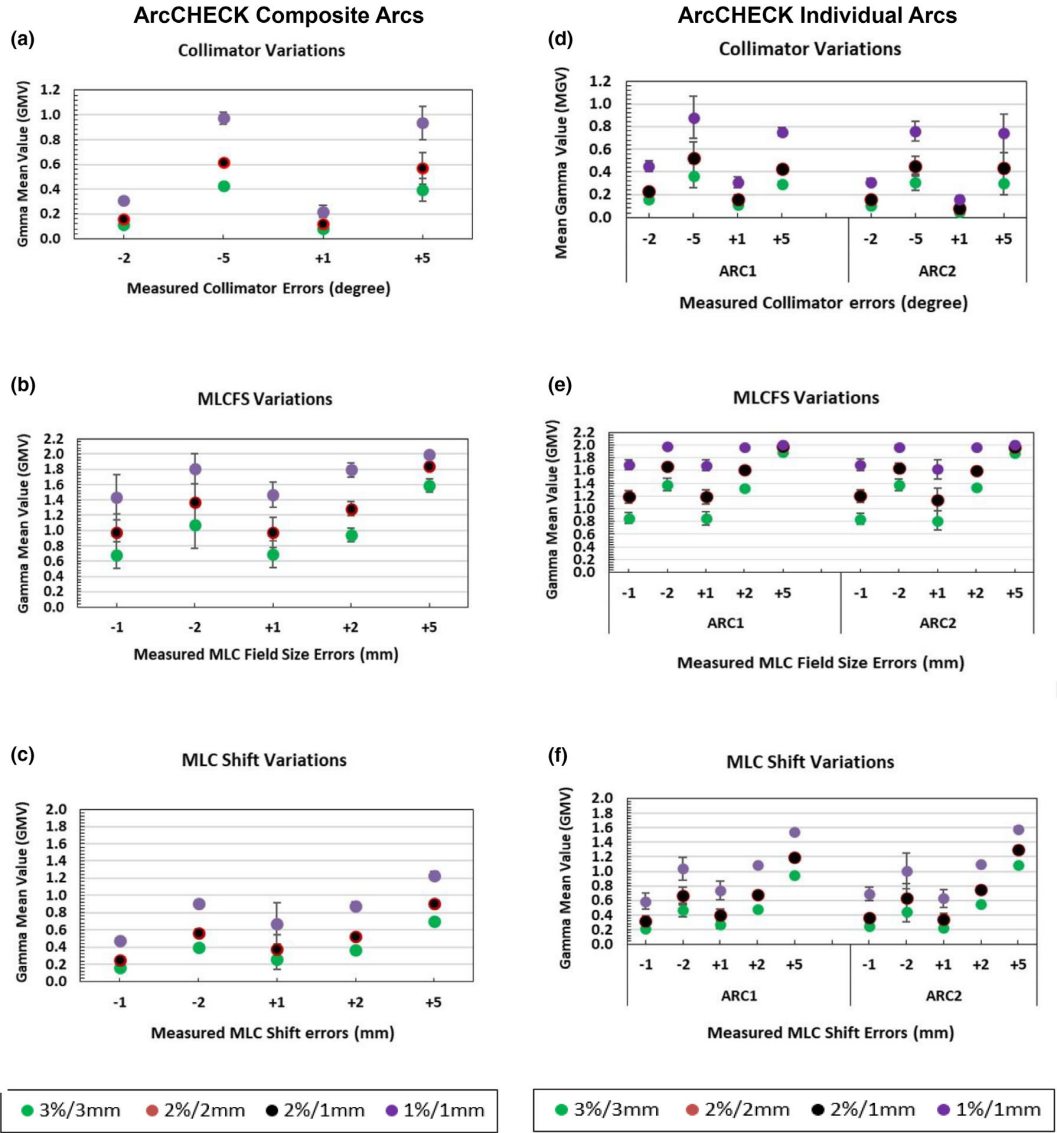
Detector intrinsic sensitivity expressed as the gamma mean values (GMV) of the measured dose distributions with introduced variations in delivery. Average mean gamma value of simulated variations in collimator and MLC when using ArcCHECK; composite arcs/plan measurements (left column; a–c) and ArcCHECK; individual arc measurement ARC1 and ARC2 (right column; d–f) with different gamma criteria. The error bars represent the standard deviation (±1SD). Correction added on May 28, 2021 after first online publication: Figure columns have been updated as ArcCHECK Composite Arcs (above left column: a‐c) and ArcCHECK Individual Arc (above right column: d‐f).

The results of the individual arc ArcCHECK measurements showed an overall pass rate lower than the composite arcs/whole plan ArcCHECK measurements (Fig. 3) and this trend was also reflected in GMV (Fig. 4). However, the error detection level was similar. For example, the errors that were not detected when using composite plan ArcCHECK measurements such as +1 and −2 collimator degrees, and ±1 mm MLC Shift were also not detected when using individual arc measurements. Again, the changes in the gamma pass rates were only seen when changing the DD% from 3% to 2% or 1% and no changes were seen when changing DTA criteria.

Further analysis of the intrinsic sensitivity of the ArcCHECK and EPID and the performance using the various gamma criteria with dose matrix resolution of 1 mm for each QA device compared to their original resolution is shown in Tables 1 and 2. Changed sensitivity was seen for variations in delivery depending on the selected gamma tolerances and on the spatial resolution of the detectors or dose matrices. For the ArcCHECK, the gamma pass rates increased by about 10 % (overestimation) when using 3%/3 mm and 2%/2 mm criteria and small changes (≤2%) were seen when using 2%/1 mm and 1%/1 mm (Table 1), whereas the EPID gamma pass rates decreased within the range of 1% −11% depending on the variation magnitude and the selected gamma criteria for each introduced variation type (Table 2). This caused some error detection changes. For example, the ArcCHECK with its original dose matrix resolution of 5 mm failed to detect some of the clinically significant errors such as +1° and −2° collimator errors and +1 mm MLC shift error when the most common 3%, 3 mm criteria were employed. The sensitivity of the ArcCHECK decreased when the dose interpolation was used and more errors were undetected, such as +1° and −2° collimator errors, ±1, ±2 mm MLC Shift. On the other hand, although gamma pass rate has changed (decreased) when converting EPID original matrix resolution of 0.244 into 1 mm but all the errors were detected when using an appropriate gamma criterion such as 2%/1 mm for the EPID in this study.

## 4 DISCUSSION

The results of the intrinsic sensitivity evaluation (comparing measured doses with errors to measured NE doses) of the ArcCHECK and EPID shows the effect of the detector resolution and different limitations between the EPID and the ArcCHECK for sensitivity to error detection in lung SBRT VMAT plans (as shown in Figs. 1 and 2).

The general sensitivity trends for both detectors agree with the findings from Maraghechi et al.,^14^ who besides comparing measured vs TPS calculated dose and TPS vs TPS, also compared measured vs measured dose using ArcCHECK and EPID for prostate plans. Generally, the intrinsic sensitivity of the ArcCHECK and EPID for the variations in the collimator and MLC shifts were similar, with decreased average gamma pass rates as the variation magnitude increased, while the ArcCHECK showed lower gamma pass rates than the EPID for the MLC field size (MLCFS) variations as those variations increased. In this case, the EPID having higher resolution might be expected to be closer to the truth and to provide lower pass rates than ArcCHECK as the errors increased. However, detector geometry and inherent characteristics of these two different detectors had a significant impact on the resulting gamma pass rate and showed that a detector with lower resolution such as the ArcCHECK may better distinguish some delivery errors.

Detector intrinsic sensitivity analysis could provide help to understand and interpret QA system results and limitations. It is clear that dose difference (DD%) controlled the gamma results for the ArcCHECK, for example, the gamma passing rates were exactly the same for 2%/2 mm and 2%/1 mm for each measured plan with variations in delivery. The ArcCHECK seems to pick up all the introduced variations except collimator variation of +1 and −2 degree. The investigated errors were selected for each plan with magnitudes that produced clinically significant errors (at a defined 2% level) and so were based on general clinical acceptability, but also these align with professional guidelines on the QA of collimator error/tolerances of approximately 1 degree.^27^, ^28^ In addition, the range of error types and magnitudes investigated for the plans (some of them being beyond the defined clinical tolerances) were selected to see what each detector can pick up and what the limitations might be for each detector considering site specific patient QA. This study considered intrinsic detector sensitivity analysis, that is, comparing detector signal with different introduced‐variations in delivery to the baseline NE plan measured signal, that is, measured vs measured. It may be noted that the ArcCHECK data showed greater sensitivity to the introduced variations than compared to the previous ArcCHECK study.^17^ In that work,^17^ the ArcCHECK‐measured error‐introduced doses were compared to the TPS calculated NE dose distributions, which is the standard clinical approach. In that previous approach the ArcCHECK was not able to detect the majority of simulated errors. On the other hand, intrinsic sensitivity analysis (measured vs measured) of the EPID and the general error detection trend is relatively comparable to the previous results (measured vs TPS calculated dose).^18^ The observed changes in the detection level and sensitivity to errors of each detector are mainly due to the underlying intrinsic sensitivity and inherent characteristics of the detector (e.g. resolution) in the first place and then also the required dose map processing (interpolation) when comparing measured vs calculated dose distributions with different resolutions in real clinical practice. The likely reason for the improved sensitivity of the ArcCHECK here, as compared to Ref. [17], is because both dose distributions (with variations and baseline, with no variations) have the same resolution and no need for interpolations (Fig. 1 and Table 1). The ArcCHECK produced pass rates largely unaffected by minor (e.g. ±1 mm shift) errors in MLC and +1, −2 degrees in collimator positioning. The results of the ArcCHECK intrinsic sensitivity analysis here can help to explain the trends reported by Woon et al.,^13^ and Maraghechi et al.,^14^ and in our previous work (ArcCHECK, measured vs TPS calculated),^17^ in which the ArcCHECK failed to detect many of the errors/variations. Weak sensitivity of the ArcCHECK detector and the impact of inherent resolution and dose matrix interpolation were also demonstrated in studies by Hussein et al.,^29^ and others.^13^, ^16^, ^30^ The low measurement (detector) resolution of the ArcCHECK and the required interpolation between measured and TPS planned dose in the clinical scenario have an impact on the sensitivity and need to be considered carefully. In comparison, the EPID gamma pass rates were more affected by changing distance to agreement (DTA) criteria and only slightly changed by changing DD% criteria as seen in Fig. 1. This trend is also demonstrated in Table 1 where there is a clear correlation between selected gamma criteria and detected variation magnitude. For example, any MLC variation of 1 mm could be detected at 2%/1 mm and not at 2%/2 mm when using the EPID. Thus, selecting appropriate gamma tolerances and criteria with each detector would improve sensitivity to the variations in delivery. The suitability of the gamma dose evaluation method in detecting clinically significant variations has been questioned in many studies.^8^, ^31^, ^32^, ^33^ However, considering a high spatial resolution detector and by eliminating other confounding factors such as interpolations, post processing of the measured and planned data and different analysis software, gamma analysis can be considered as a meaningful and informative tool as seen from the EPID intrinsic sensitivity analysis in this study.

The results in Fig. 2 for the GMV support results from the GPR and show similar trends for the ArcCHECK and EPID. The GMV in Fig. 2 shows the mean and spread of the gamma score (±1SD) for each error type, increasing as the gamma pass rate decreased for each error. The trend at 2%/2 mm and 2%/1 mm gamma tolerances illustrates a perfect agreement for the ArcCHECK data for all introduced‐variation types and a disagreement for the EPID data as the DTA tolerance has changed and hence so has the detection sensitivity. Low resolution detectors, such as ArcCHECK could pick up dose differences but can miss geometric errors due to the impact of resolution limitations to the application of the DTA criterion, whereas the high resolution EPID was able to pick up errors via the DTA criterion.

The results of the composite ArcCHECK measurements were repeated on individual fields (arc‐by‐arc) to confirm the previous results and to reduce potentially confounding variables. Some previous studies have suggested that field‐by‐field approaches may be more stringent than for composite measurements, since the latter may mask some errors.^7^, ^31^, ^32^, ^34^, ^35^ Those studies were mostly referring to field‐by‐field at gantry 0 and or collapsed composite measurements. Although lower pass rates were observed in the current work for the individual arc ArcCHECK measurements, than for the composite measurements, the error detection levels were similar for the lung SBRT VMAT plans investigated here.

The results for the EPID and the ArcCHECK were further supported by assessing the effect of the detectors’ dose matrix resolution on the gamma pass rate as seen in Tables 1 and 2. Both dose matrix sampling resolutions were changed to 1 mm for the ArcCHECK (oversampled) and the EPID (under sampled) relative to their original resolution to illustrate the effect of the spatial resolution with and without interpolation. By comparing detector signals (measured plans with variations to the measured baseline plans), the ArcCHECK gamma pass rate increased by about 10% and the EPID gamma pass rate dropped by 1–11% depending on the selected gamma tolerance and variation magnitude. The observed trend was a result of the interpolation and resampling, which could happen in a real clinical situation when interpolation is required to compare TPS planned dose to the measured dose with different detector resolution. This also may result in potential false positives, or false negatives, in error detection and reduce confidence in the QA outcomes, as demonstrated by Bailey et al.,^36^ and Hussein et al.,^29^ Therefore, the choice of gamma tolerances should be specific for each clinical treatment site and detector used to ensure accurate dose verification, as recommended in AAPM TG 218.^7^ The optimal detector is one which would identify significant differences between delivered and calculated doses. For this, it is important to understand the intrinsic properties of the system and test its different components independently where possible to establish proper tolerances and optimize QA analysis protocols. Intrinsic detector sensitivity analysis, especially in terms of reported gamma results, can provide a baseline optimal sensitivity to be expected from any system using a given detector type. It could also be recommended as part of local system commissioning tests to help understand and interpret QA system results.

The intrinsic detector sensitivity analysis in this study not only confirmed the effect of the detector resolution on the gamma analysis but also demonstrated the impact of the selected gamma criteria and dose matrix interpolation in the clinical setting when using sparsely distributed (low resolution) detectors. This study together with our previous work^17^, ^18^ has also highlighted some points to consider when making a sensible decision for an optimal detector for a specific clinical site and to avoid using a detector beyond its intrinsic limits. This could also help explain the apparently differing findings in the literature, regarding relative ArcCHECK and EPID detection sensitivity, as discussed above. It is likely that if the introduced variations (errors) in delivery resulted in significant changes in the dose (e.g. changes in DVH metrics) and the DD% tolerance used for the gamma analysis was appropriate, then the error would be detected by the ArcCHECK, particularly for complex plans like H&N, whereas for other combinations of error type and gamma tolerances this might not be the case. For the EPID, differences in detection sensitivity between different studies can also likely be explained by the differences in the combination of measurement method, EPID dose model, dose calculation algorithm and protocols and the tolerances and thresholds used. Therefore, a passing gamma calculation may hide a clinically significant dose deviation according to the plan, when using low detector resolution with inappropriate gamma criteria selection, especially DD%. On the other hand, this could also happen with high spatial resolution detectors such as the EPID here, when the gamma criteria are insufficient to detect a specific error magnitude or dose deviation, especially selecting DTA. The original inherent detector characteristics along with any required interpolation in the real clinical scenario can play a major role to determine the sensitivity of each detector in specific delivery/plan conditions.

The effect of criterion choice on the gamma analysis was reported by Nelms et al.,^37^ showing that DTA value can significantly affect the sensitivity of the gamma analysis to detect errors. Woon et al.,^13^ raised concern as to whether this will only occur when using low spatial resolution detectors and stringent gamma criteria of 2%/2 mm and 1%/1 mm. Our findings showed that DTA value affected both low and high spatial resolution detectors in different ways, over the range of common gamma criteria of 3%/3 mm, 2%/2 mm and more strict criteria of 2%/1 mm and 1%/1 mm. Maraghechi et al.,^14^ besides those same four, also investigated 3%/1 mm, showing it was more sensitive than the other criteria when using ArcCHECK and EPID for prostate plans. Specifically for lung SBRT VMAT plans, Saito et al.,^11^ found that the Delta 4 (Scandidos) and the PTW 2D array were not sensitive to small MLC errors using gamma criteria of 3%/3 mm, 3%/2 mm and 3%/1 mm and 2%/2 mm. They also suggested using DD% alone as a more useful tool than using gamma analysis. This again supports our discussion regarding the insensitivity of low spatial resolution detectors and the domination of the DD% metric in the gamma analysis and not the DTA. Additionally, Kim et al.,^38^ investigated the sensitivity of EBT2 films and MapCHECK to detect MLC misalignments and found that the most common criterion of 2%/2 mm was not sufficiently sensitive and recommended 2%/1 mm to evaluate VMAT plans for SBRT techniques. Our studies showed that 2%/1 mm seems to be appropriate for lung SBRT VMAT plans when using a high spatial resolution detector.

This study using intrinsic detector sensitivity analysis is a proof of concept of such an analysis tool to consider for further future work, using various detectors with different configurations. It could potentially be used by detector manufacturers to validate detector sensitivity and aid detector characterization for clinical use in a consistent way.

These findings emphasize that gamma analysis results should be carefully considered and interpreted using appropriate tolerances. They also emphasizes the importance of understanding intrinsic characteristics and limitations of each detector.

## 5 CONCLUSION

This study investigated intrinsic sensitivity of the ArcCHECK and EPID as a method to characterize detectors and their sensitivity to detect variations in delivery of lung SBRT VMAT plans. The findings demonstrated the effects of detector resolution and dose matrix interpolation on gamma analysis results and highlighted different limitations between EPID and ArcCHECK. Care is needed in selecting gamma criteria and tolerances, particularly when interpolation is required. The selected comparison criteria (DTA and DD), in combination with the detector resolution, can significantly affect resulting gamma pass rates for this application. DTA criteria had a higher impact on the gamma result when using the EPID and a strong correlation was seen between selected DTA and detected variations. Conversely for the ArcCHECK, the DD% criteria impacted the resulting gamma pass rates. Thus, high spatial resolution detectors could pick up changes in the field and variations in delivery for these SBRT treatments using appropriate DTA, while a low spatial resolution detector would pick up the variations using DD% if they affect the dose, whilst the sensitivity was not much affected by changes to DTA criteria. The detector intrinsic sensitivity approach can explain the intrinsic limits of each detector and provide a baseline limiting sensitivity. This could help in the selection of more meaningful gamma criteria and the optimal QA device for site‐specific dose verification.

## CONFLICT OF INTEREST

The authors declare no conflict of interest.
